# Tumour hypoxia in clinical oncology: imaging, therapeutic strategies, and translational challenges

**DOI:** 10.1016/j.ctro.2026.101279

**Published:** 2026-09-07

**Authors:** Xuehua Lin, Maitiú Ó. Murchú, Luke Sheridan, Soumya Menon, Gerard G. Hanna, Helena M. Kelly, Christopher R. Simpson

**Affiliations:** a School of Pharmacy and Biomolecular Sciences, RCSI, Ardilaun House, 111 St Stephen's Green, Dublin, Ireland; b Department of Surgery, Trinity St. James's Cancer Institute, Trinity Translational Medicine Institute (TTMI), Trinity Centre for Health Sciences, Trinity College Dublin, Dublin, Ireland.; c Discipline of Radiation Therapy, Trinity Centre for Health Sciences, Trinity College Dublin, Dublin, Ireland.; d St Luke's Radiation Oncology Network, St James's Hospital, Dublin, Ireland.

**Keywords:** Radiosensitisation, Hypoxia, Clinical trials, Tumour oxygenation

## Abstract

Hypoxia, a defining feature of many solid tumours, arises from rapid cellular proliferation, abnormal tumour stroma, and aberrant vasculature. Reduced oxygen availability contributes to several cancer hallmarks, including sustained proliferative signalling, angiogenesis, metabolic reprogramming, immune evasion, invasion, and metastasis. Consistent with these biological effects, tumour hypoxia is associated with poorer prognosis across multiple cancer types, irrespective of treatment modality. Consequently, the characterisation and therapeutic leverage of hypoxic tumour status have been key areas of clinical investigation over the past three decades. However, despite extensive research, no method for routine clinical stratification of tumours by hypoxic status has been widely adopted, and hypoxia-targeted or hypoxia-alleviating treatments remain limited in clinical use.

This review summarises the clinical progress and ongoing challenges in hypoxia imaging and therapeutic intervention. It focuses on strategies designed to enhance oxygen delivery, exploit hypoxic tumour environments through hypoxia-activated prodrugs, or modify tumour oxygen consumption. By these approaches under clinical evaluation, this review aims to identify key barriers to the integration of hypoxia-focused intervention and highlight the volume and diversity of hypoxia targeted treatments that are pending clinical translation.

## Highlights

• PET/CT imaging and MRI can identify hypoxic tumours but require standardisation.
• Clinical nimorazole use highlights the need for hypoxia-based patient selection.
• Direct tumour reoxygenation strategies may improve tumour radiation response.
• Certain hypoxia-activated prodrugs may require improved delivery and formulation.
• Reducing tumour oxygen consumption is a new therapeutic strategy to reduce hypoxia.

## 1 Introduction

Solid tumours present a heterogeneous structure of aggregated stroma, blood vessels, immune cells, fibroblasts, signalling molecules and extracellular matrix. These cellular and noncellular components form the tumour microenvironment (TME), which governs tumour survival, growth, angiogenesis, invasion, metastasis, colonisation and treatment-resistance [1], [2]. In solid tumours, features of the TME, including abnormal vasculature, dense stroma and uncontrolled cell proliferation, can culminate in hypoxia, an oxygen-starved state [3]. Direct measurements of oxygenation in human tumours has demonstrated lower oxygen levels than in normal tissues, with reported median partial pressure of oxygen (pO_2_) ranging from approximately 0.3% - 4.2% (2.0–32.0 mmHg) in tumour, compared with 3.4% - 6.8% (26.0–51.6 mmHg) in normal tissues [4], [5]. However, it should be noted that physiological pO2 varies according to tissue type, and tumour hypoxia can be generally recognized as a departure from the levels of directly adjacent normal tissue. Moreover, hypoxia within the TME is spatiotemporally heterogeneous, and can present as chronic, acute and cyclic states [6].

Chronic hypoxia, or diffusion-limited hypoxia, presents during tumour development, typically lasting from a few hours to many days [6]. This form of hypoxia arises due to rapid proliferation of cancer cells, increasing cellular volume that in turn extends diffusion distances between cells and nearby blood vessels [7]. Accordingly, tumour cells proximal to functional blood vessels receive sufficient oxygen, whereas increasing distance from the vasculature can result in diffusion-limited oxygen supply [8]. Recent in vivo imaging studies have demonstrated an inverse correlation between distance from the nearest vessel and tissue oxygenation across several animal tumour models, although the strength of this correlation varied between tumour types [9]. In addition to tumour types, oxygen diffusion distance is also influenced by the vascular geometric differences, blood flow direction and vascular diffusion shunts [5] In contrast, acute hypoxia, or perfusion-limited hypoxia can occur near to vessels as a result of transient fluctuations in blood flow associated with aberrant angiogenesis or irregular red blood cell flux, demonstrated in experimental tumour models [5], [10], [11], [12], [13]. Acute hypoxia usually lasts from minutes to hours, and reversed with restoration of adequate blood flow and oxygen supply [7]. Repeated fluctuations in oxygen availability, with periods of hypoxia followed by reoxygenation, are referred to as cyclic hypoxia. This dynamic state exhibits complex temporal behaviour which can induce varied downstream consequences. For instance, rapid cycles can occur, ranging from 2 to 5 cycles per hour have been reported across multiple tumour types, including canine and human cancers, which may be driven by thermoregulation [14], whereas slow cycles occur from hours to days and have been proposed to arise from a longer term physiological event, vascular remodelling [15].

**Fig. 1:**
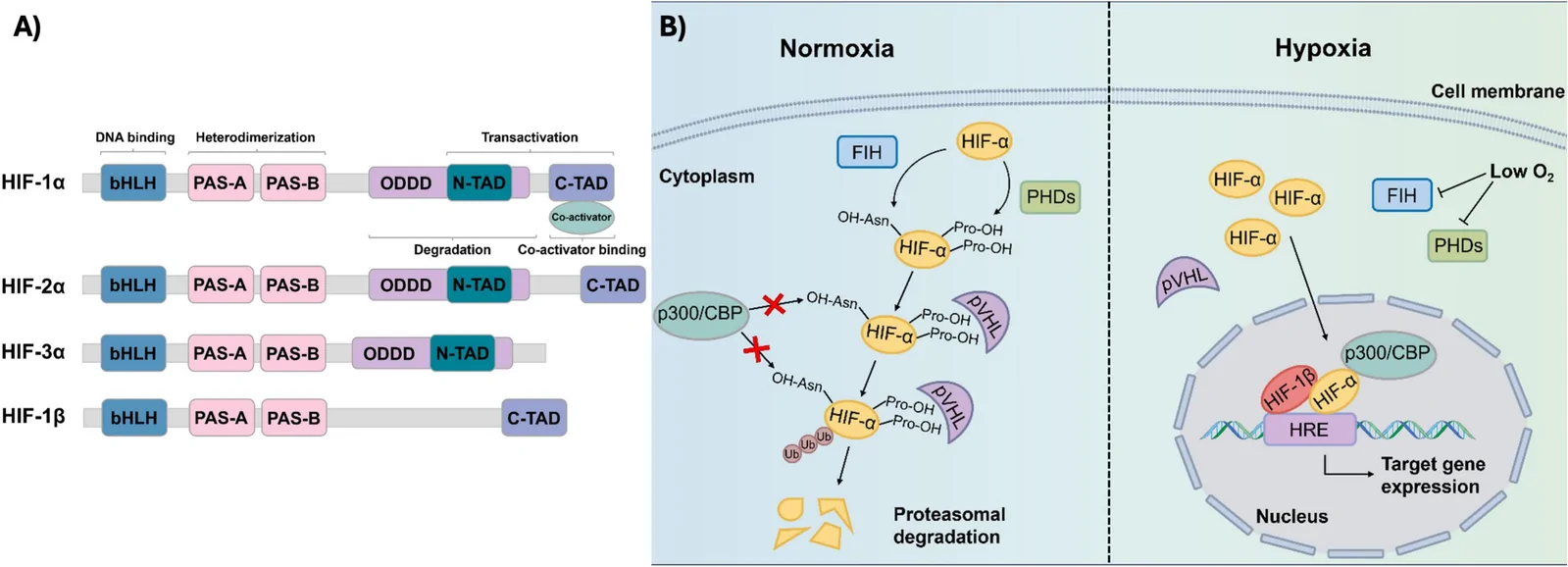
HIF isoforms share conserved DNA-binding and dimerisation domains but differ in their transactivation domains and transcriptional programmes. B) Under normoxic conditions, the oxygen-dependent degradation (ODD) domain of HIF-α is rapidly hydroxylated by prolyl hydroxylase domain proteins (PHDs), promoting interaction with von Hippel–Lindau (VHL) protein and subsequent proteasomal degradation. Under hypoxic conditions, HIF-α is stabilized and translocated to the nucleus, where it heterodimerizes with HIF-1β, recruits p300 and CREB-binding protein (CBP), and binds hypoxia response elements (HREs) within target genes to activate HIF-α-dependent transcriptional programmes. Figure adapted from Shi et al., HIF-1 and HIF-2 in cancer: structure, regulation, and therapeutic prospects, Cellular and Molecular Life Sciences, Springer Nature, published 18 January 2025 [16].

Exposed to dynamic and complex hypoxia profiles, cancer cells adapt through the activation of hypoxia-inducible factors (HIFs), a family of transcription factors that serve as master regulators of hundreds of hypoxia-responsive genes. HIFs are heterodimeric DNA binding complexes composed of hypoxia-responsive subunits (HIF-1α, HIF-2α and HIF-3α) and a constitutive HIF-1β/aryl hydrocarbon receptor nuclear translocator subunit (Fig. 1) [16]. HIF-1α and HIF-2α both possess basic helix-loop-helix and Per–ARNT–Sim (PAS) domains that mediate DNA binding and heterodimerisation, an oxygen-dependent degradation domain (ODDD), and N- and C-terminal transactivation domains. HIF-3α is understood to have multiple splice variants with context-dependent functions, including modulation of HIF-1- and HIF-2-mediated transcription. Under hypoxia, reduced oxygen-dependent hydroxylation stabilises HIF-α subunits, enabling their nuclear accumulation, heterodimerisation with HIF-1β, recruitment of transcriptional co-activators including p300/CBP, and binding to hypoxia-response elements within target genes [16].

Importantly, cellular response to hypoxia is not restricted to HIF signalling. HIF-independent responses include suppression of mechanistic target of rapamycin (mTOR) signalling and activation of the unfolded protein response (UPR), which both alter protein synthesis, metabolism, autophagy and cell-survival programmes during oxygen deprivation. The details of these hypoxia induced biochemical mechanisms and pathways are extensive and broad, and have been reported in greater detail elsewhere [17].

**Fig. 2:**
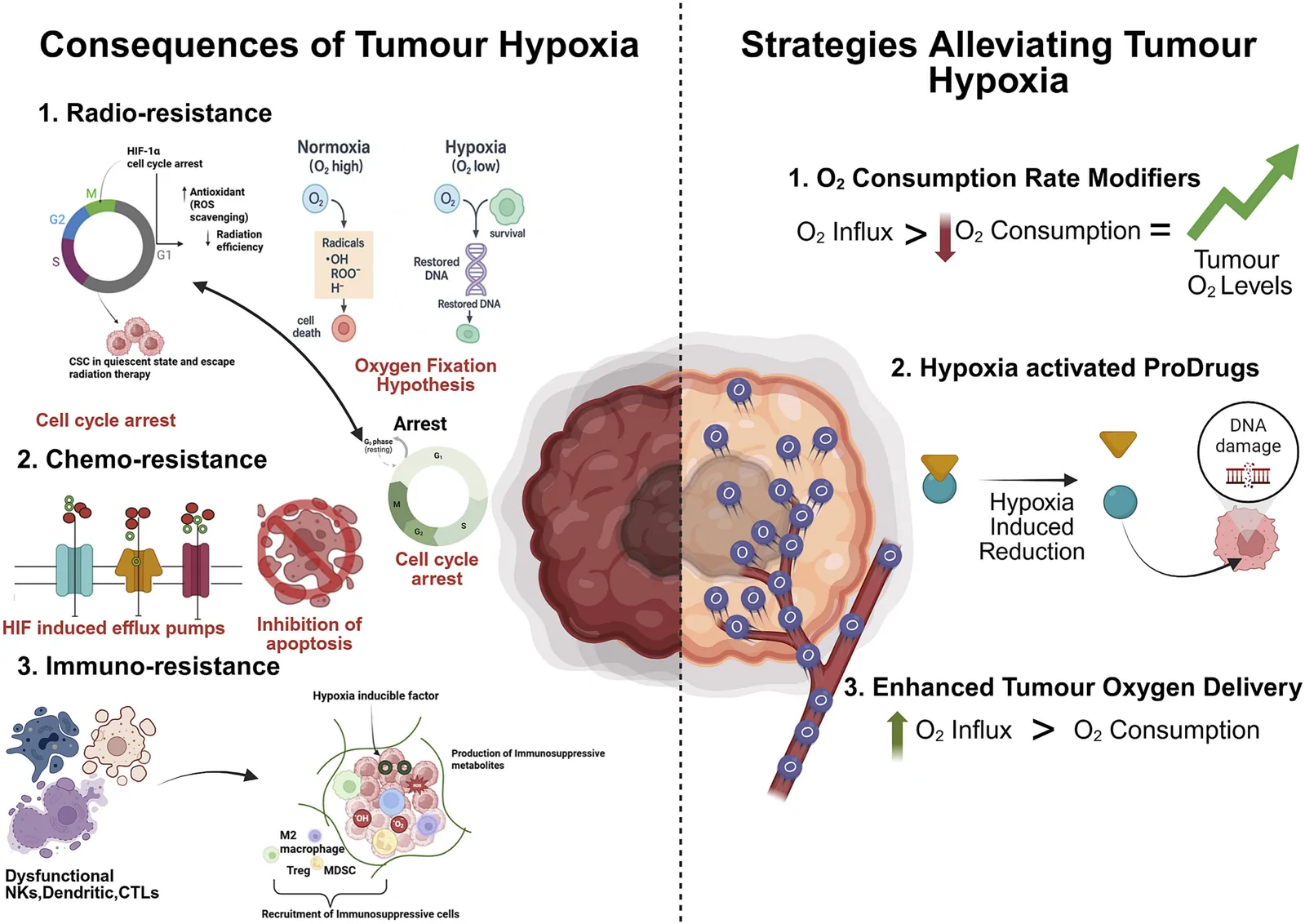
Activation of hypoxic pathways has been implicated across radio, chemo and immunotherapy resistance. To counteract hypoxia's role in resistance and disease progression new strategies that alleviate or leverage the hypoxic environment are being developed.

Hypoxia-induced cellular adaptations promote resistance to both radiotherapy and chemotherapy (Fig. 2) [18], [19], [20], [21], [22]. In solid tumours, chemotherapy failure is often associated with acquired multidrug resistance (MDR), which emerges through genetic and epigenetic alterations, with hypoxia as a key driver [23]. Specifically, resistance can arise through:

1. *Hypoxia regulated TME*: Aberrant angiogenesis, elevated interstitial fluid pressures, and dense fibrotic stroma hinder drug delivery and drug residence [24].
2. *HIF-induced efflux pumps*: Upregulation of drug resistance genes, increasing efflux transporters such as MDR1/P-glycoprotein, MRP1 and BCRP, expelling chemotherapeutic drugs from cancer cells [25].
3. *Inhibition of apoptosis*: HIF-regulated genes suppress apoptosis; for instance, HIF-1α reduces p53 activity, limiting 5-FU-induced cell death in gastric cancer [26].
4. *Cancer stem cells (CSCs)*: Hypoxic niches maintain CSC quiescence and enhance survival via Epithelial-Mesenchymal Transition (EMT), MDR, dormancy, cell-cycle arrest and improved DNA repair [27], [28].

Beyond chemotherapy, the association between hypoxia and treatment resistance is also well established in radiotherapy. Radiotherapy resistance in hypoxic tumours has been recognized since 1953, when Gray and colleagues showed that oxygen present at the time of irradiation is required for maximal radiosensitivity, and that hypoxic cells require approximately threefold higher radiation doses to achieve equivalent cell death [29]. Mechanistically, this effect has been explained by:

1. *The Oxygen Fixation Hypotheses*: In the presence of oxygen, radiation-induced DNA radicals react with molecular oxygen, promoting the formation of chemically stable, “fixed” lesions. Under hypoxic conditions, oxygen-dependent fixation is reduced, allowing radiation-induced radical damage to undergo chemical restoration [30], [31], [32].
2. *Cell Cycle arrest:* Hypoxia can induce cell cycle arrest, particularly in the G0/G1 and S phases, through activation of several hypoxia-responsive checkpoint pathways. Radiosensitivity varies across the cell cycle, with cells in G0/G1 and S phases generally exhibiting lower radiosensitivity that those in G2/ M phases. Consequently, hypoxia-induced cycle arrest may maintain cells in relatively less radiosensitive states, thereby contributing to radioresistance [33].
3. *Anti-Oxidant production*: Under hypoxic conditions, cancer cells exhibit elevated reactive oxygen species (ROS) levels, resulting in increased oxidative stress [34]. In response, cancer cells can upregulate antioxidant defences to buffer ROS and mitigate ROS-induced oxidative damage. This enhanced antioxidant capacity may also limit the accumulation of radiation-induced oxidative damage, which may contribute to radioresistance [35].

Lastly, hypoxia resistive mechanisms are also an emergent contributor to immunotherapy outcomes. This has been demonstrated by hypoxic regions recruiting immunosuppressive immune cells, including regulatory T cells (T_reg_), myeloid derived suppressor cells (MDSCs), M2 macrophages, thus enhancing an immunosuppressive phenotype [36], [37]. Simultaneously, hypoxia contributes to the dysfunction of other immune response cells such as on cytotoxic T lymphocytes (CTLs), dendritic cells (DCs), natural killer cells (NKs). HIFs also alter the metabolic landscapes producing immunosuppressive metabolites, such as ROS, adenosine and kynurenine, which further hinders the effectiveness of infiltrating immune cells within tumours [38].

### 1.1 Clinical imaging of tumour hypoxia

Despite clinical evidence linking pretreatment tumour hypoxia with poor outcome and treatment resistance, translating hypoxia assessment into routine clinical decision-making remains challenging. A major barrier is the lack of a fully validated biomarker capable of capturing the spatial and temporal heterogeneity of clinically relevant hypoxia. Direct pO_2_ measurements using polarographic electrodes such as the Eppendorf probe provided evidence for the clinical significance of hypoxia, but their invasive, operator-dependent nature and restriction to accessible tumour sites have limited broader adoption. Consequently, current efforts have focused on non-invasive biomarkers, particularly hypoxia positron emission tomography (PET), with magnetic resonance imaging (MRI) based approaches emerging as promising complementary proxy measurements. These methods may support prognosis, treatment-response assessment and patient stratification for hypoxia-targeted interventions, but they are not yet standardized for routine use across tumour types.

PET was first introduced as an imaging tool in cancer in the early 1990s and is based on the use of gamma ray photons to produce an image [39]. For hypoxia capture, PET compatible radioisotope tracers have been designed to specifically accumulate in hypoxic regions after bioreduction [40]. Nitroimidazole derivatives are the most extensively studied hypoxia PET tracers in the clinical setting. For this review, our analysis of registered and published clinical studies, 18F-fluoromisonidazole (FMISO) and 18F-fluoroazomycin arabinoside (FAZA) were the two most frequently evaluated tracers, with FMISO reaching phase III clinical evaluation. Other tracers that have been less extensively clinically investigated include HX4, EF5, Cu-ATSM and selected gallium- or technetium-labelled agents. Accordingly, this review focuses on FMISO and FAZA, while broader overviews of alternative hypoxia tracers are available elsewhere [40], [41], [42]. Later-generation radiotracers are also emerging in preclinical studies, in some cases with improved pharmacokinetics and tumour-to-background contrast but will require further clinical evaluation.

FMISO is a lipophilic nitroimidazole tracer that preferentially accumulates in hypoxic cells, where limited oxygen availability prevents reoxidation of reduced metabolites, leading to intracellular trapping. FMISO has been well established for its ability to detect tumour hypoxia in head and neck [43], [44], [45], breast [46], [47] and lung cancers. Increased FMISO uptake in gliomas has been associated with tumour expression of hypoxic biomarkers, including carbonic anhydrase-IX (CA-IX), as well as angiogenesis markers including Vascular Endothelial Growth Factor (VEGF), and has also been correlated with poorer overall survival outcomes. Similarly, recent studies in treatment naïve breast cancer patients, showed greater tumour FMISO uptake to correlate with increased CA-IX expression and smaller vessel diameters [46]. Further study into FMISO uptake in breast cancer also reveals subtype associations, with oestrogen and progesterone negatives having higher signals of hypoxia and poorer prognosis [47]. These findings are also consistent with studies in head and neck cancers with greater FMISO hypoxia signal correlated with poorer progression free survival (PFS) and overall survival (OS) [43].

The utility of FMISO-guided treatment adaptation has also been demonstrated in HPV-related oropharyngeal carcinoma [48]. In a phase II study, patients who underwent primary tumour resection and were treated with radiotherapy for residual nodal disease, underwent baseline FMISO PET hypoxia imaging. Patients without evidence of hypoxia at baseline or during treatment received de-escalated chemoradiotherapy to 30 Gy, whereas those with persistent hypoxia received standard treatment to 70 Gy. Disease control and survival were high in both cohorts, with reduced toxicity in the de-escalated, non-hypoxic groups group. This study further confirmed that persistent intra-treatment FMISO-defined hypoxia was associated with a 3.5-fold increased risk of distant metastasis and poorer overall survival, while baseline hypoxia-negative patients remained free from distant metastasis [49]. However, as treatment allocation was determined by hypoxia status and was non-randomized, these data support the feasibility of FMISO-guided adaptation but requires confirmation from a randomized validation study.

Overall, the utility of FMISO as a tumour hypoxia indicator has shown clinical research value, but larger prospective studies are still needed to validate clinically actionable thresholds, establish sensitivity and specificity for hypoxia assessment, and standardize imaging-guided decision-making. In addition, FMISO has practical limitations, including slow clearance from normoxic tissue, delayed imaging requirements, and limited image contrast [40].

Consequently, a second-generation tracer FAZA, was developed and first evaluated clinically for hypoxia imaging in 2009 [50], showing increased hydrophilicity, low lipophilicity, increased vascular clearance, and greater hypoxia–normoxia contrast [51]. A 2012 clinical trial further validated FAZA's effectiveness in detecting tumour hypoxia and its prognostic value in head and neck cancer. Following radiotherapy, patients with hypoxic tumours, as defined by FAZA presence, had a disease-free survival rate of 60%, compared to 93% in those with normoxic tumours at 19 months follow up [52]. The clinical use of FAZA has also been further evaluated in hypoxic imaging of gliomas, rhabdomyosarcomas and as well as lung, head, neck, cervical and rectal tumours [52], [53], [54], [55], [56], [57], [58], [59].

**Fig. 3:**
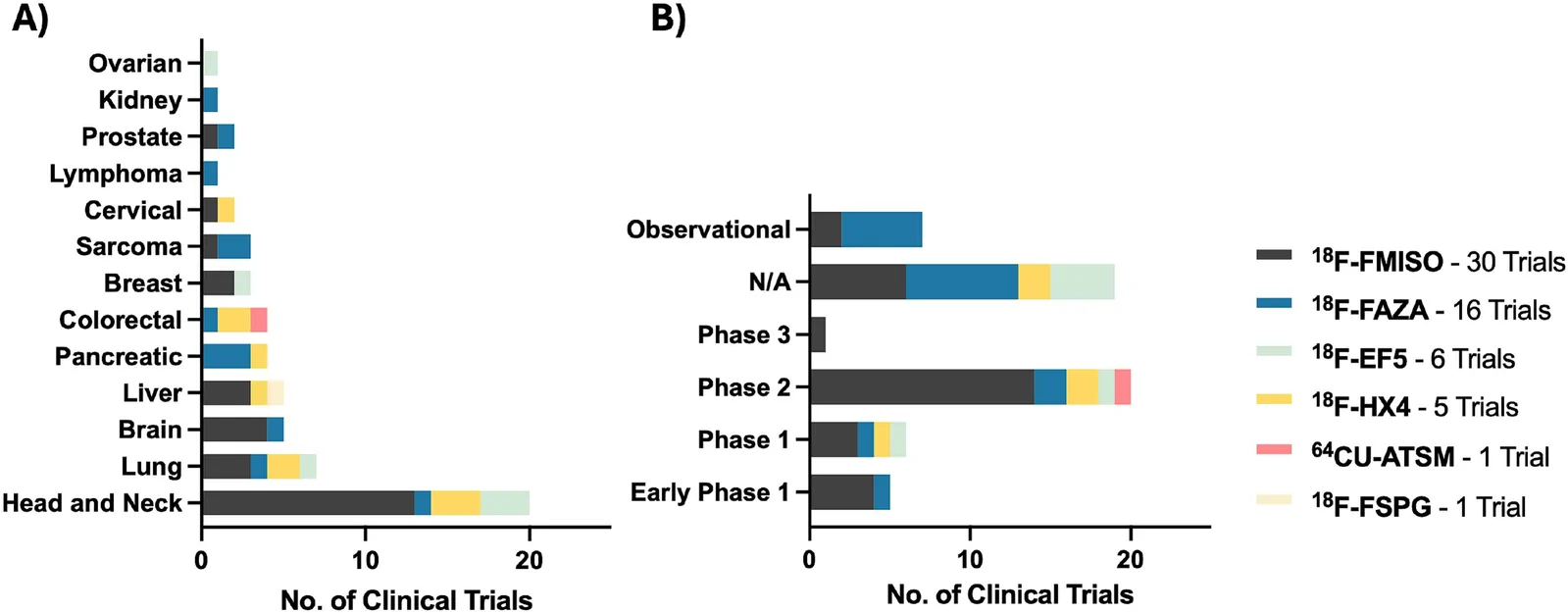
Clinical trials using radiotracers for imaging hypoxia on clinicaltrials.gov. A) Number of clinical trials by cancer type. B) Number of trials using radiotracers by study type. Trials were categorized as completed, recruiting, not yet recruiting or active, not recruiting.

In parallel to the ongoing work on the use of PET to evaluate tumour hypoxia, MRI has also been evaluated in clinical trials. MRI uses a strong magnetic field to force protons in the body to align with the magnetic field. A radiofrequency current is then used to generate images with contrast determined by differences in magnetic properties between tissue types. MRI is a more widely available and lower cost technique than PET and it also does not require the use of radiotracers, making it a more practical option for the clinical setting [40]. Current MRI techniques used in hypoxia evaluation are focused on oxygen enhanced MRI (OE-MRI) and include blood oxygenation level dependent (BOLD) and tumour oxygenation level dependent (TOLD) techniques. BOLD is based on the ability of MRI to detect differences between oxygenated and deoxygenated haemoglobin, therefore producing signals that reflect blood oxygen saturation. However, blood oxygenation is not necessarily representative of tissue oxygenation, in particular when dealing with avascular tumours, which may limit its application. TOLD requires patients to inhale 100% oxygen or carbogen to induce arterial hyperoxia and then relies on differing characteristics between oxygenated and deoxygenated regions to produce a signal that reflects oxygen saturation within tissues. This approach relies on hyperoxygenation having a limited effect on hypoxic areas of tissue which has been validated in a number of in vivo studies [60]. There is a broad range of pre-clinical studies validating this approach across a range of cancer types [61]; however, translation to the clinic has, to date, been limited. The small number of clinical studies using OE-MRI include head & neck, glioblastoma, hepatocellular and NSCLC [62], have shown the clinical feasibility of OE-MRI to identify hypoxic regions in tumours [63], [64], [65]. However, these studies are small, single institute trials and therefore there are interinstitutional differences due to a lack of standardisation of protocols in terms of imaging sequences and timing. Validation of OE-MRI in larger, multicentre trials is required to enable the use of as a clinically relevant biomarker [61], [62]. Currently a number of clinical trials are undertaking comparative studies of PET and MRI in specialised centres, which will add to the body of evidence relating to MRI, however there is a continued lack of multicentre trials to provide the required data to support widespread clinical implementation (Fig. 3).

### 1.2 Therapeutic strategies for targeting clinical hypoxia

As hypoxia imaging has been developing, there has in parallel been ongoing work to develop effective therapeutic strategies to circumvent hypoxia. This section focuses on hypoxia-targeted treatment modalities that have reached clinical trial and assesses the current evidence.

### 1.3 Nimorazole

Nimorazole (NIM) is the only true hypoxic radiosensitizer that is used as part of clinical practice. NIM is 5-nitroimidazole compound with electron affinity which functions as an oxygen mimetic [66]. The use of this molecule is restricted to Danish practice, where it is applied with curative chemo- radiotherapy in larynx (not stage I gliotic), pharynx, oral cavity and sinonasal cavity squamous cell carcinomas. The evidence base for Denmark's clinical recommendation stems from a series of clinical trials coordinated by The Danish Head and Neck Cancer Group (DAHANCA), **including subsequent international collaborative studies** [67]. DAHANCA-5 demonstrated a significant improvement in locoregional control with NIM compared with placebo (49% NIM vs. 33% placebo, *p* = 0.002) with an associated improvement in disease-specific survival but no statistically significant overall survival benefit [68]. This was subsequently investigated in the international, multicentre EORTC-1219/DAHANCA-29 phase III trial, combining NIM with moderately accelerated radiotherapy (IMRT, 70 Gy, 6 weeks) and cisplatin, achieving significantly increased locoregional control in advanced HPV-negative HNSCC compared to placebo (3 year locoregional failure: 20.7% NIM vs 33.8% placebo) [69]. However, again the use of NIM did not significantly improve patient overall survival (62.4% NIM vs 61.5% placebo). AHANCA's latest focus regarding NIM is refining treatment allocation to hypoxic patients most likely to benefit. DAHANCA 30 has completed recruitment, and early analyses suggest that patients expressing a *less-hypoxic* 15-gene expression profile may safely omit nimorazole, with no apparent detriment in locoregional control. Although final mature results are still awaited, these results inform the latest NIM guidelines published by DAHANCA [67].

Outside Denmark, the evidence base for NIM is more heterogeneous. For instance, IAEA-HypoX reported improved locoregional control with accelerated radiotherapy; however, low patient recruitment meant the study was underpowered and it has since closed [70]. NIM featured as the intervention in the UK NIMRAD phase III trial with IMRT in patients unsuitable for platinum chemotherapy or cetuximab. This study found no significant improvement in locoregional control or overall survival. This also included patients categorized as hypoxic using a gene-expression profile. Hence, in the context of IMRT for older and less fit patients with hypoxic tumours, NIM's clinical benefit was less evident [71]. It is possible that because of the mixed evidence NIM is yet to be clinically adopted elsewhere.

### 1.4 Hyperbaric oxygen therapy (HBOT)

HBOT is primarily used to treat decompression sickness of divers resurfacing too quickly [72]. This treatment involves housing patients in a chamber purged with 100% oxygen at an increased pressure (above 1 atm absolute). Recompression helps reduce inert gas bubble size, promoting elimination, while the high oxygen levels improve tissue oxygenation to prevent further injury [73], [74].

The ability of HBOT to induce higher tissue oxygenation has been functionally extended to reversal of hypoxia in solid tumours. This has been specifically reported in head and neck cancer where HBOT achieved increases of pO_2_ of up to 550 mmHg, with hypertoxic status lasting for 5–25 min after chamber decompression [75]. Similarly, in glioblastoma tissues, raised pO_2_ levels of approximately 45 mmHg and 65 mmHg were respectively reported in the intra- and peritumoral regions after HBOT. This hyperoxic status remained for 15 min after leaving the hyperbaric chamber [76]. The improvement in radiosensitisation arising from hypoxia reversal in head and neck and glioma has since been clinically demonstrated as reported in Table 1.

**Table 1:** Clinical evidence of HBOT and radiation reported in head and neck cancers and gliomas.

**Evidence type**	**Cancer type**	**Year**	**Study/design**	**Result**	**Limitations**
Radiosensitisation	HNSCC	1999	Randomized HBOT + radiotherapy trial [77]	Positive tumour-control outcomes reported	Older treatment protocols; radiation fractionation differs from modern practice
Radiosensitisation	Laryngeal SCC	1999	Hypo-fractionated radiotherapy + HBOT [78]	Long-term tumour control reported	Confounding by hypofractionation; late toxicity
Modern RT combination	HNSCC	2010	Phase I HBOT + chemo-IMRT dose-escalation study [79]	Feasible without dose-limiting toxicities	Phase I; not powered for efficacy
Modern RT combination	HNSCC	2017	Phase I dose-escalation HBOT + IMRT + chemotherapy [79]	Safe/tolerable; complete clinical responses reported in completers	Small cohort; controlled efficacy trial required
Modern RT combination	Glioblastoma	2017	HBOT + IMRT + multi-agent chemotherapy [80]	Median OS 22.1 months vs 17.2 months with comparator regimen	Small cohort; local progression in >50% of treatment arm
Stereotactic RT	Recurrent high-grade glioma	2007	HBOT + fractionated stereotactic radiotherapy using gamma unit [81]	Survival benefit reported in recurrent anaplastic astrocytoma and GBM	Small cohort
Stereotactic RT	Recurrent high-grade glioma	2021	HBOT + hypo-fractionated stereotactic radiotherapy [82]	Low toxicity; 55.5% disease control at 3 months	Small cohort
Stereotactic RT	Brain metastases	2022	Proof of concept HBOT immediately before SRS; 19 HBOT-treated patients compared with 19 matched historical controls [83]	Feasible and well tolerated; favourable trends in clinical outcomes, but no statistically significant improvement compared with controls	Small, non-randomized study using historical controls, not powered to demonstrate efficacy
Hypoxia modification / systemic therapy	Advanced/metastatic gastric or gastro-oesophageal junction adenocarcinoma	2024–2026 (ongoing)	Prospective single-arm Phase Ib/II trial of XELOX + sintilimab + HBOT; planned *n* = 57 [84]	Recruiting; no efficacy results yet. HBOT is being investigated as a strategy to modulate tumour hypoxia and enhance treatment response	Ongoing, single-arm study, no clinical efficacy results yet; does not include radiotherapy

HBOT with radiotherapy has shown less promising clinical results in bladder [85] and cervical cancers [[86], [87]]. No clinical evidence of benefits in bladder cancer have been observed in terms of local control, mortality, local recurrence and quality of life after incorporating HBOT [88]. With regard to cervical carcinoma, the evidence reporting HBOT efficacy in radiosensitivity is heterogenous. A Medical Research Council Trial evidenced that HBOT improved local control and survival, specially benefiting patients under age 55 who presented with Stage III carcinoma of uterine cervix [86]. However, other clinical trials have reported no benefit in tumour control, 1- or 5-year mortality, and increased side effects with the use of HBOT for patients with cervical tumours [[86], [87], [89], [90], [91]].

While HBOT has shown promise for head and neck cancer and high-grade gliomas, a high prevalence of severe late complications has been noted in trials of head and neck cancer [[77], [92], [93]]. Current clinical trials are focused on the incorporation of modern radiation treatment regimens, which may help reduce complications and enhance treatment response. More recently, HBOT has been investigated as a strategy to enhance responses to immune checkpoint inhibition by alleviating tumour hypoxia, with an ongoing phase Ib/II trial evaluating HBOT in combination with XELOX and a PD-1 inhibitor in gastric and gastro-oesophageal cancers [84]. However, it must be acknowledged that practical barriers to the widespread clinical implementation of HBOT remain, as radiotherapy should begin within the tumour oxygenated timeframe [[94], [95]] creating workflow and infrastructure challenges.

### 1.5 Carbogen breathing

Carbogen, a mixture of carbon dioxide (CO_2_) and oxygen (O_2_), has served as a standard of care for carbon monoxide poisoning (COP) for a half century. Carbogen is normally administered by mask or endotracheal tube, which increases the mobility of patients relative to HBOT. Compared to HBOT, carbogen also increases intratumoural oxygen diffusion, counteracts issues of vasoconstriction and is better tolerated for patients with severe co-morbidities [[96], [97]]. Carbogen was initially composed of 5% CO_2_ and 95% O_2_, however a 2% CO_2_ mixture has been clinically demonstrated to show comparable efficacy to 5% CO_2_ with improved compliance, making it widely accepted in clinical trials [98].

ARCON [accelerated radiation (AR)] with carbogen and nicotinamide (NTM)) [99], is a promising hypoxia-modifying strategy with an aim of counteracting radioresistance. NTM is a modified vitamin which helps to increase blood flow in tumours and thereby serves as a radiation sensitizer [99]. ARCON has been established as a safe regimen with manageable side effects, and has achieved therapeutic improvements in head and neck, and bladder cancers both in combination and as a monotherapy. With regard to head and neck cancer, ARCON has yielded high local and regional control rate in two phase III clinical trials [[100], [101]]. In one phase III randomized study in advanced laryngeal carcinoma (NCT00147732) patients were further stratified based on pimonidazole-assessed tumour hypoxia. After ARCON treatment, improvements in regional control were observed in the hypoxic patient cohort relative to patients with well‑oxygenated tumour (100% vs 55%, respectively; *P* = 0.01) [101]. A phase III study in 2013 measured the median expression of 26-gene signatures to assign a Hypoxia Score (TLDA-HS) for the selection of patients with hypoxic or less hypoxic laryngeal cancer and bladder cancer patients. Laryngeal cancer patients scoring a high hypoxia score, gained a better 5-year regional control rate [102]. In contrast, TLDA-HS scores did not indicate benefits for ARCON treatment in bladder cancer.

In comparison other research does show ARCON's effectiveness in bladder cancer management in a series of clinical trials [[103], [104], [105], [106], [107]]. A randomized phase III trial in 2010 indicated that significant differences in OS, risk of death, and recurrence were in favour of ARCON treatment when compared to radiation alone [105]. A further study in 2017 focused on patient stratification, investigating the prognostic and predictive ability of a hypoxic 24-gene signature. In the radiation alone arm, patients categorized as more hypoxic by a high 24-gene score had poorer local-progression-free survival (LPFS) than those categorized as less hypoxic. More interestingly, patients with high-hypoxia scores receiving ARCON had an improved LPFS rate than those undergoing RT alone [107]. ARCON however is not a “one size fits all” approach It has shown no clear benefit in glioblastoma multiforme [[108], [109]] and non-small cell lung cancers [110].

### 1.6 Trans‑sodium crocetinate

Trans‑sodium crocetinate (TSC) is a first-in-class, small molecule designed to re‑oxygenate deprived tissue. TSC enhances oxygenation of tissue by improving its diffusion through water. The alteration in movement kinetics is not a direct interaction of TSC and oxygen, but TSC's rearrangement of water molecules into a more organised structure [111]. This change in the water molecule behaviour in plasma or interstitial fluid enables faster oxygen movement from blood and through tissue [112]. The potential use of this synthetic compound was initially to treat haemorrhagic shock from blood loss after severe trauma [113], with subsequent studies evaluating its use in peripheral artery disease (PAD).

Of more relevance to tumour hypoxia, TSC has received an orphan drug designation from the United States Food and Drug Administration for glioblastoma multiforme (GBM). In this context, TSC was tested in a Phase II trial as a radiosensitizer in newly diagnosed GBM patients. 50 patients were given a bolus intravenous dose of TSC (0.25 mg/kg) 3 times a week 45 min before radiation therapy [114]. For the entire 6 weeks of radiotherapy, before standard of care (temozolomide) was started. The trial showed TSC to be safe with survival at 2 years was 36% after the intervention compared to historical data indicating survival rates at 26–30%, a modest improvement. Since 2021 there have been no further records of TSC in clinical study for tumour hypoxia or radiosensitisation purposes.

### 1.7 PFCs-based oxygen-carrying colloids

Perfluorocarbons (PFCs) are organofluorinated compounds with high oxygen solubility, enabling them to dissolve and transport comparatively more oxygen than water or serum and to passively release it along diffusion gradients in hypoxic tissues [[115], [116], [117], [118], [119]]. Early PFC emulsions were investigated as artificial blood substitutes in the 1980s and 1990s, but high systemic dose requirements, toxicity concerns, and formulation limitations prevented clinical translation [[117], [120]]. PFC emulsions have also been explored as tumour oxygenation agents to enhance radiotherapy response. This was initially reported preclinically with products Flousol-DA and Oxygent showing radiosensitisation of tumour models when administered with carbogen breathing [[121], [122], [123], [124]].

Beyond this, the clinical translation of PFC-based hypoxia alleviation has been limited. A Phase I/II trial initiated in 1987 evaluated Fluosol-DA plus 100% oxygen adjunct to conventional radiotherapy in 18 patients with high-grade brain tumours [[125], [126]]. Treatment was associated with low rates of serious toxicity, but median survival was not significantly improved overall: 75 weeks in the Fluosol-DA group versus 54 weeks in controls. Further subgroup analysis did show a survival benefit favouring Fluosol-DA using the Gehan–Wilcoxon test, although this finding requires cautious interpretation due to potential bias due to the subgroup consisting of patients that survived beyond one year [[125], [126]].

More recent clinical interest has focused on dodecafluoropentane emulsion (DDFPe), originally developed as an ultrasound contrast agent and subsequently repurposed by NuvOx Pharma as a radiosensitiser, NVX-108/NanO_2_™ [127]. A Phase Ib study in newly diagnosed glioblastoma evaluated NVX-108 with oxygen/carbogen breathing alongside standard chemoradiation. The dose-escalation study identified 0.1 mL/kg as the recommended dose and reported no acute NVX-108 toxicity. TOLD MRI showed significant tumour hypoxia reversal without significant oxygenation changes in normal brain tissue, supporting selective tumour oxygenation. Clinical outcomes were also encouraging, with reported median PFS of 9.6 months and median OS of 19.4 months compared with historical temozolomide plus radiotherapy outcomes [[128], [129]]. At the time of writing, the Phase II RESTORE trial (NCT03862430) is evaluating NanO_2_™ versus saline in combination with standard radiotherapy and temozolomide in newly diagnosed GBM, with NanO_2_™ administered intravenously before daily radiation to test whether pharmacological tumour oxygenation can improve radiotherapy efficacy.

### 1.8 Hypoxia-activated prodrugs

Hypoxia-activated prodrugs (HAPs) are designed to exploit the hypoxic tumour microenvironment by undergoing enzymatic activation in oxygen-deficient regions to generate cytotoxic species [[130], [131]]. Several HAPs, including tirapazamine, evofosfamide and PR-104, have progressed to late-phase clinical trials.

### 1.9 Tirapazamine

Tirapazamine (TPZ) was the first reported HAP in 1986. It is bio-activated to a cytotoxin by one-electron transfer under moderately hypoxic conditions. The resulting oxidizing radical anion produces the lethal double-strand break in DNA. In addition, it may accentuate DNA damage induced by radiation or DNA-damaging agents [[132], [133]]. Despite promising results from Phase I/II trials when TPZ was IV administered in combination with cisplatin and/or radiation for advanced head and neck cancer, cervical and lung cancer, subsequent phase III trials of TPZ failed to show a survival benefit and also presented concerns about toxicity, resulting in the clinical development of TPZ being put on hold [[133], [134], [135], [136], [137]]. The cause of clinical failure of TPZ is multifaceted, one of which is that some tumours do not harbour a sufficiently large population of hypoxic cells, meaning the expected effect of TPZ may have been overestimated in these cases [135].

Recently, loco-regional delivery of TPZ via chemoembolization has been designed to activate TPZ exclusively in the tumour, and in parallel to reduce potential systematic toxicity. A phase I clinical trial of TPZ followed by superselective trans-arterial embolization (TAE) in patients with unresectable hepatocellular carcinoma (HCC) demonstrated no significant adverse effects (AE) with encouraging tumour response, justifying pursuit of phase II clinical trial (NCT02174549) [138]. Conversely, in locally advanced cervical cancer, a Phase III Gynaecologic Oncology Group trial comparing standard cisplatin-radiotherapy with cisplatin + TPZ + radiotherapy showed no significant improvement in survival, indicating limited added value in that context [133]. Earlier Phase I studies combining weekly TPZ with radiation (external beam + brachytherapy) and cisplatin in cervical cancer showed acceptable toxicity but did not translate into superior outcomes in later trials [139]. These findings suggest TPZ effectiveness may be context- and delivery-dependent, with localized hypoxia (as in embolized HCC tumours) potentially offering a more favourable therapeutic window than systemic approaches in cervical cancer.

### 1.10 Evofosfamide (TH-302)

Evofosfamide (TH-302) is a second-generation hypoxia-activated prodrug that reduces to release the cytotoxic DNA-alkylating Br-IPM mustard under hypoxic tumour conditions [140]. Compared to TPZ, TH302 is activated at relatively lower oxygen concentrations, so only severely hypoxic tumour cells within tumours may be targeted by TH-302. Across 11 xenograft models, TH-302 shows high selectivity for hypoxic fractions, with hypoxic volume strongly correlated to total anti-tumour effect [[140], [141]]. TH-302 has yielded promising results up to phase II trials when used as monotherapy and in combination therapy with traditional chemotherapy drugs, including doxorubicin, gemcitabine and bevacizumab [[142], [143], [144], [145]]. However, it was less successful in a phase III trial where advanced/unresectable/metastatic soft-tissue sarcoma patients, were given either doxorubicin alone or doxorubicin with TH-302. This trial reported limited efficacy and multiple associated toxicities [146] possibly due to lack of patient stratification based on tumour hypoxic levels. Therefore, the use of predictive biomarkers of hypoxia maybe useful in further clinical investigations [144] [147].

Recent clinical trials (Table 2) evaluating TH-302, demonstrate its growing therapeutic relevance across multiple hypoxic cancers, including pancreatic, prostate, and oesophageal tumours. Notably, emerging data from the newly initiated Phase I/II trial (NCT06782555) highlights the integration of TH-302 with immune checkpoint inhibitors (zalifrelimab and balstilimab) in advanced solid tumours, suggesting a shift in focus, toward overcoming tumour hypoxia–driven resistance and enhancing immunotherapeutic efficacy.

**Table 2:** Clinical trials of evofosfamide in solid tumours. Overview of completed, terminated, withdrawn, planned, and ongoing studies evaluating evofosfamide alone or in combination with immunotherapy, chemotherapy, radiotherapy,

**Trial (NCT)**	**Cancer Type / Setting**	**Phase & Design**	**Intervention**	**Key Findings**	**Status**
NCT06782555	Castration-resistant prostate cancer, pancreatic cancer, HPV-negative head & neck squamous cell carcinoma	Phase I/II, open-label, dose-escalation + expansion	Evofosfamide (IMGS-101) + zalifrelimab (anti-CTLA-4) + balstilimab (anti-PD-1)	First patient dosed early 2025; evaluates safety, tolerability, PK, and preliminary antitumor activity in advanced solid tumours; hypoxia-targeted immunotherapy approach	Recruiting, primary completion January 2028
NCT02342379	Glioblastoma (recurrent, bevacizumab-refractory)	Phase II, single-arm, dual-centre	Evofosfamide 670 mg/m^2^ + bevacizumab	4-month PFS: 31% vs historical ∼3%; median OS 4.6 months; PR 9%, SD 43% [145]	Completed 2021
NCT03098160	Prostate, pancreatic, melanoma, HNSCC	Phase I, dose-escalation, in combination with ipilimumab	Evofosfamide (400–640 mg/m^2^ d1,8) + ipilimumab (3 mg/kg d8)	No new safety signals; PR in 17%, SD in 67%; immune gene signatures predict response	Completed 2019
NCT02598687	Oesophageal cancer (preoperative)	Phase I, combination with carboplatin and paclitaxel, radiotherapy	Evofosfamide + carboplatin/paclitaxel + RT	Withdrawn early (low enrolment)	Withdrawn 2016
NCT01864538	Advanced melanoma	Phase II, biomarker-enriched	Evofosfamide monotherapy	Trial terminated; no outcome posted	Terminated 2015
NCT02047500	Pancreatic cancer	Phase I combination with gemcitabine/nab-paclitaxel	Evofosfamide + *gem*/nab-paclitaxel	Terminated; no detailed data	Terminated 2016
NCT06836726*(EVO)*	Castrate-resistant prostate cancer post-ARSI	Phase II	Evofosfamide + continued Androgen receptor signalling inhibitors (ARSI)	Not yet recruiting (launched 2025)	Planned 2029

### 1.11 PR-104

A further HAP drug that progressed to clinical trials was PR-104. PR-104 undergoes hydrolysis and then one- and/or two-electron reduction, finally generating active cytotoxins capable of crosslinking DNA and inducing apoptosis [131]. PR-104 can target not only hypoxic cells but also aerobic cells, generating a cytotoxic effect. One-electron reduction, governed by cytochrome 450 oxidoreductases (POR) accounts for the majority of activity of PR-104 in hypoxic conditions, while oxygen-insensitive two-electron reduction governed by aldo-keto reductase 1C3 (AKR1C3), accounts for its activity in aerobic conditions [131]. Across in vivo preclinical models, PR-104 has demonstrated anti-tumour activity as single agent against a range of hypoxic tumours [148] or/and the aerobic fraction of tumour expressed AKR1C3 [149], and greater effects when used in combination with chemotherapy (e.g. gemcitabine/docetaxel [148] and sorafenib [150]) or/and radiation therapy [148]. However, several phase I clinical studies on PR-104 either as monotherapy or with sorafenib or chemotherapy, have reported no objective tumour response and dose-limiting myelotoxicities (e.g. thrombocytopenia and neutropenia) [[151], [152], [153], [154]]. It is hypothesised that toxicity occurred via mechanisms that may involve the metabolic activation of hypoxic niche or/and AKR1C3 in normal bone marrow, followed by DNA-crosslinking to blood progenitor cells [154]. This toxicity profile has prompted research on the activity of PR-104 in the treatment of acute lymphoblastic leukaemia (ALL) and acute myeloid leukaemia (AML), where the leukemic microenvironment is markedly hypoxic and therefore can be a possible target for PR-104. A phase I/II study of PR-104 monotherapy, demonstrated clinical response in 10 out of 31 AML patients and 2 out of 10 ALL patients [155], suggesting potential for further investigation in these indications. No new late-phase trials have been initiated as of the most recent clinical registry updates, indicating that PR-104's future clinical development may depend on targeted patient selection (e.g., AKR1C3 biomarker-guided) or safer prodrug reformulations.

### 1.12 Apaziquone

**Apaziquone (EO9)** is a prodrug, that converts into an active DNA damaging agent, in turn inducing apoptosis. Activation of EO9 can occur through different bio reductive mechanisms and the type of activation is highly dependent on environmental conditions. These mechanisms are extensively covered elsewhere, however, pertinent to hypoxia, the bioreductive enzyme NQO1 is the most studied due to the clear oxygen sensitive processing of EO9 [[156], [157], [158], [159], [160], [161]].

Despite favourable data from pre-clinical and a Phase I E09 monotherapy trial [162], evaluation of EO9 in Phase II studies across a range of cancers including NSCLC, pancreatic, breast, colorectal and gastric cancers showed no activity [[163], [164]]. Possible reasons for this included EO9's rapid pharmacokinetic elimination after systematic circulation [[162], [165]] as well as EO9's poor penetrating ability through multiple cell layers [166] resulting in poor delivery and subsequent efficacy. To overcome this, intravesical administration of E09 as monotherapy, was evaluated in further Phase I& II trials which reported good safety profiles and significant anti-tumour activity against bladder cancer patients [[167], [168], [169]]. The success of these phase I/II and phase II trials [NCT00461591 and NCT00598806] [131] paved a way for two large, multi-centre phase III trials with a total of 1746 patients with bladder cancer which failed to yield a statistically significant response. An expert opinion on the initial phase III trials highlights differences in study design between the phase II and III trials and suggested that the lack of biomarker stratification (NQO1 or hypoxic status) could be significant in determining efficacy [170]. As of writing this review the planned phase III trials for EO9 in bladder cancer have since been terminated. However, investigations into radiosensitivity has been observed a variety of preclinical hypoxic tumours after treatment of irradiation in combination with EO9 [160] suggesting EO9 could have a role here, but there hasn't been any apparent translational effort into radiotherapy trials since this preclinical data.

### 1.13 Banoxantrone

**Banoxantrone (AQ4N**) is an advanced aliphatic N-oxide with a history of uncertainty and mixed evidence supporting its use. AQ4N possesses no DNA binding activity under normoxia, however, through cytochrome P450 isozymes (CYPs) or nitric oxide synthase 2 A- mediated reduction, under hypoxic conditions, activation of AQ4N to AQ4 results in a 1000-fold cytotoxic potency compared to the prodrug form, which can deeply penetrate within tumour tissues, whilst the prodrug is cleared from the circulation [[171], [172]]. Despite promising pre-clinical data [[172], [173], [174], [175]], Phase I results have been mixed. Notably, AQ4N was also evaluated in combination with fractionated radiotherapy in a Phase I study of patients with oesophageal cancer, where tumour concentrations of the active metabolite AQ4 exceeded those in normal tissue, supporting hypoxia-selective activation in the clinical setting [176]. Plasma concentrations of bioreduced AQ4N active metabolites were appreciably lower or even undetectable, causing no objective anti-tumoral activity demonstrated in the open-label phase 1 dose-escalation study by Papadopoulos et al. [177]. Other phase I trials have however shown that the concentration of AQ4N exceeded active levels in preclinical models with no dose- limiting toxic effects [[141], [176], [178], [179]]. However no clinical development of AQ4N has progressed further than phase II trials. Current research efforts are more focused on **preclinical studies** exploring novel delivery methods, such as nanoparticles, to overcome the pharmacokinetic and drug delivery issues that plagued earlier trials. This suggests that while the concept of AQ4N remains of interest, its path to widespread clinical use is currently on hold, awaiting more effective delivery strategies [[180], [181]].

In summary, despite extensive investigation, up to and including Phase III clinical trials, no HAP is currently approved for routine use in the treatment of hypoxia and trial results have offered conflicting results on their efficacy [182]. This failure of major HAP clinical trials may be reflected in the lack of effective biomarkers or imaging modalities to identify patients most likely to benefit from HAP therapy [147]. This hypothesis is supported by retrospective studies, when patients were stratified based on tumour hypoxia biomarkers, therapeutic benefit was demonstrated within hypoxic groups, despite overall trial failure [[183], [184]].

## 2 Therapy modifying oxygen consumption rate (OCR)

### 2.1 Metformin

Metformin's potential as an anti-cancer agent was identified after the observation was made that diabetic patients taking metformin had a lower cancer incidence than patients with or without diabetes taking other medicines [[185], [186], [187], [188]], which was subsequently supported by retrospective studies. The mechanisms through which metformin modulates tumour growth is reported to be bifold, either working indirectly to reduce insulin and glucose within the circulation, through reduced gluconeogenesis or directly through the inhibition of complex I of the Electron Transfer Chain. This inhibitory action leads to a reduction in ATP production with the overall result leading to reduced oxygen consumption rate (OCR), and thus improved tumour oxygenation [[130], [189]]. In addition, glucose availability influences nutrient-sensing pathways such as PI3K/AKT/mTOR, which can modulate HIF-1α-dependent adaptation, suggesting that metformin may affect tumour hypoxia not only through reduced respiration but also through altered metabolic signalling [[190], [191]].

However, metformin's performance across clinical trials demonstrated conflicting results [[192], [193], [194]]. A recent meta-analysis evaluated 22 randomized clinical trials across a variety of tumour types including reproductive (breast, ovary, endometrium, and prostate), respiratory (lung), and digestive (pancreas and liver) system cancers. These studies evaluated metformin at doses of 500–2000 mg daily [195]. The results indicated that metformin treatment was not associated with reduced cancer-related mortality in adults compared with placebo or no treatment. However, it did show potentially beneficial effects on the PFS of the patients with reproductive system cancers but was related to a worse PFS in patients with digestive system cancers.

The differential response observed between cancers could be due to stratification by hypoxic status. The potential importance of such stratification was demonstrated in a randomized phase II study in locally advanced cervical cancer (NCT02394652). In this trial, only patients showing detectable tumour hypoxia using FAZA PET scans were enrolled. After one week of treatment, metformin reduced hypoxic volume by an average of 10.2%, in contrast the hypoxic volume increased by 4.7% in the control group [196]. Although the study was terminated early and included only 13 hypoxic patients, these findings suggest in principle, that metformin can modify tumour hypoxia. A second randomized phase II study, (NCT04275713), evaluated metformin as a hypoxia-modifying treatment alongside cisplatin based chemoradiotherapy in locally advanced cervical cancer [197]. Metformin 850 mg was given twice daily, one week before radiotherapy, with subsequent MRI and tumour biopsies assessing hypoxia related changes. The combination was well tolerated, although no significant change was seen in the primary hypoxia gene-expression signatures, yet greater tumour-volume reduction was observed in the metformin arm.

The clinical evaluation of metformin so far, highlights several limitations for conclusive efficacy in hypoxia modification, including heterogeneity in tumour type, dose, combination treatment, eligibility criteria and, importantly, limited stratification according to tumour hypoxia. While metformin has not demonstrated consistent benefit across unstratified cancer populations, hypoxia-focused cervical cancer studies suggest that its activity may be more easily identified when patients are selected or evaluated according to the metabolic phenotype targeted by metformin. Therefore, hypoxic biomarkers could be key in establishing the clinical evidence base for metabolic modifiers.

More generally strategies that reduce cellular oxygen consumption rates of cells near blood vessels and within more well perfused tissue within the tumour, may efficiently alleviate hypoxia within the hypoxic niche by increasing oxygen concentration within the tumour microvasculature, but also through the reduction of the hypoxic gradient within the tissue itself [[130], [198]]. This strategy can be used to increase sensitivity to radio and chemo therapies; and has been demonstrated in both xenograft and in vitro models [[189], [199], [200]]. Targeting hypoxia in this manner offers several advantages compared to HAPs including not being reliant upon diffusion to be transported into poorly vascularised hypoxic fractions which allows for application to a broader range of cancer types [130].

Phenformin, another biguanide and more potent inhibitor of mitochondrial respiration than metformin, has also been proposed as a strategy to reduce tumour oxygen consumption [[130], [199], [201]]. Preclinical studies demonstrate reductions in OCR and hypoxic radioresistance at lower concentrations than metformin [188]; however, its ability to alleviate tumour hypoxia has not been evaluated clinically, possibly due to safety concerns regarding its association with severe lactic acidosis, which has led to its withdrawal from clinical use in certain countries [202]. Yet, phenformin has had recent clinical trial interest as a metabolic adjunct to BRAF/MEK inhibition, with the aim of enhancing treatment response and delaying resistance in melanoma [203]. Nevertheless, phenformin may warrant further clinical investigation as an OCR-targeting strategy, particularly in prospectively selected patients with hypoxic tumours, who are most likely to benefit.

### 2.2 Atovaquone

The anti-malarial agent atovaquone, has been reported to reduce OCR by up to 80% across a large range of cancer types through inhibition of ECT complex III [[130], [204], [205]]. Atovaquone has a strong safety profile with ideal pharmacokinetics and low cost, making it a highly appealing candidate with potential to reduce tumour hypoxia. Ashton et al. (2016) demonstrated that atovaquone decreased hypoxic tumour environments in 3D spheroids and this could be illustrated to decrease spheroid regrowth following irradiation treatment ex vivo and in xenograft models; showing greater potency than both metformin and phenformin [[130], [205]].

Early clinical data has further demonstrated promising results. In a phase I trial, atovaquone has been associated with a reduction in hypoxia-regulated genes highlighting that targeting tumour mitochondrial metabolism could reduce hypoxia leading to antitumor effects at an mRNA level, in NSCLC [206]. An additional focus of this research was to image the spatial changes in oxygenation as a result of atovaquone. The treatment appears to result in the greatest reduction in hypoxia, within the most hypoxic central tumour regions, which would originally be associated with the greatest resistance to treatment [207]. Following on from this, a phase I trial (NCT04648033) has recently reported that atovaquone, up to a dose of 750 mg twice daily, was safe alongside chemoradiotherapy. Moreover, in concordance with earlier trial data, clinical FMISO PET-CT scans showed a dose dependent lowering of hypoxia levels recorded in tumours with atovaquone treatment. With that said, the phase I trials used a small patient cohort of 21 patients. The next step of this research is to evaluate the potential of atovaquone in improving lung cancer treatment, while possibly increasing cohort sizes. More recent clinical trials are assessing atovaquone in paediatric gliomas with radiotherapy (NCT06624371) [208] and in platinum-resistant ovarian cancer (NCT05998135) [209].

## 3 Conclusions

The clinical significance of hypoxia in cancer patient prognosis and survival has been long realised, propelling interest into imaging techniques to identify hypoxic areas in solid tumours. Growing research in hypoxia imaging, particularly with PET tracers such as FMISO and FAZA, demonstrate momentum toward larger, multi-cancer clinical trials that may ultimately enable translation into routine practice. Other stratification techniques that are appearing include the identification of hypoxia-targeting biomarkers, or tumour-type-dependent hypoxia-associated gene signatures. However, widespread adoption will require not only further validation but also the development of standardized procedures for grading and reporting spatial tumour hypoxia. The establishment of these procedures will be essential for initiating and guiding clinical practice toward treating hypoxic tumours.

Denmark has led on targeting hypoxia with radiosensitisers with its clinical adoption of NIM as a standard intervention in HNSCC, however it remains the only country using this approach. The most recent clinical focus of this treatment is now refining benefit to tumours characterised as hypoxic, but a uniform stratification method is still lacking, highlighting the need for decisive and accessible imaging modalities or genetic screening to define patient cohorts likely to benefit.

**Fig. 4:**
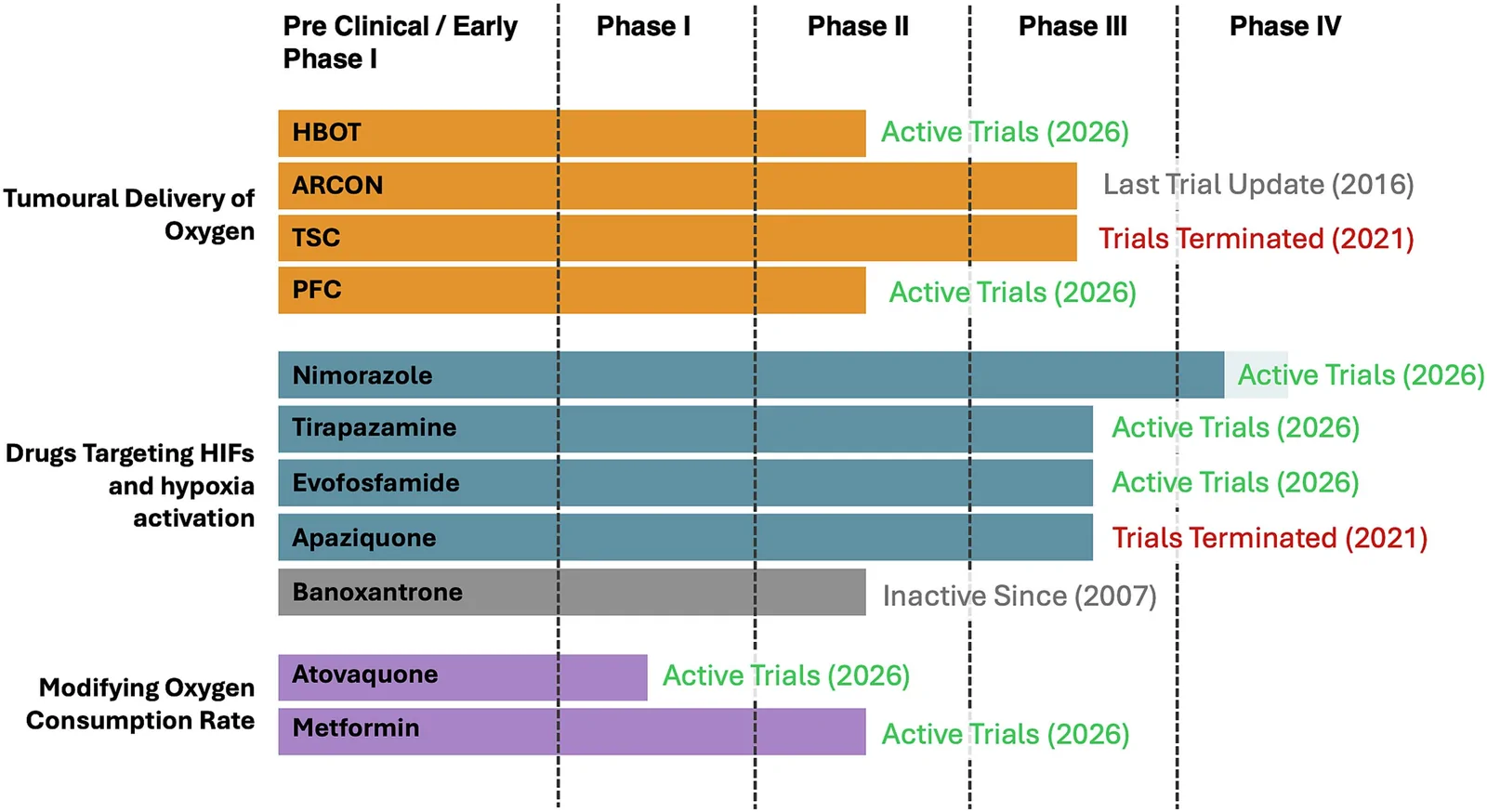
Progression summary of strategies that target tumour hypoxia. Showing maximum clinical phase achieved and current status in clinical trials. Clinical trial phases correct as of August 2024.

Other tumour hypoxia therapeutic strategies remain diverse and out of widespread clinical practice (Fig. 4). Increasing oxygen delivery to tumours, can enhance radiosensitivity however, these treatments are non-specific to the tumour and can be further limited by the abnormal and inefficient vasculature that creates hypoxia in the first instance. More specific approaches in targeting hypoxia by leveraging the low O_2_ environment to activate drugs or targeting HIF pathways offer promising treatment options that could improve standard-of-care outcomes and mitigate treatment resistance. Because of this, therapies such as evofosfamide are increasingly appearing alongside approved immunotherapies. However, drug distribution of these therapies to hypoxic regions can be problematic via systemic administration suggesting efficacy could be optimised with study of dosage regimens, delivery strategies and formulation design. This is particularly evident from apaziquone, showing potential in direct delivery to the bladder in bladder cancer patients**.** An emerging but less mature strategy focuses on reducing tumoural oxygen consumption rates, with early clinical evidence suggesting the alleviation of specific hypoxic regions. While randomized trials are needed to confirm the therapeutic relevance of these effects on radiosensitivity or chemoresistance, the reliance on repurposed, clinically approved drugs may accelerate translation of the medicines used in this approach.

## CRediT authorship contribution statement

**Xuehua Lin:** Writing – review & editing, Writing – original draft, Investigation, Conceptualization. **Maitiú Ó. Murchú:** Writing – review & editing, Writing – original draft, Investigation, Conceptualization. **Luke Sheridan:** Writing – review & editing, Writing – original draft, Investigation, Conceptualization. **Soumya Menon:** Writing – review & editing, Visualization, Investigation. **Gerard G. Hanna:** Writing – review & editing. **Helena M. Kelly:** Writing – review & editing, Writing – original draft, Supervision, Funding acquisition, Conceptualization. **Christopher R. Simpson:** Writing – review & editing, Writing – original draft, Visualization, Supervision, Conceptualization.

## **Informed consent**

Not applicable

## **Ethics approval**

Not applicable

## **Author agreement**

All authors have reviewed and approved the final revised version of this manuscript. The manuscript is original, has not been published elsewhere, and is not under consideration for publication elsewhere.

## **Funding**

The salaries of the first and corresponding authors for this review were supported by the Royal College of Surgeons in Ireland’s, Strategic Academic Recruitment programme (StAR), under grant numbers 21393A08 and 24550A001, respectively.

## Declaration of competing interest

The authors declare that they have no known competing financial interests or personal relationships that could have appeared to influence the work reported in this paper.
